# Transgenic Mosquitoes Expressing a Phospholipase A_2_ Gene Have a Fitness Advantage When Fed *Plasmodium falciparum*-Infected Blood

**DOI:** 10.1371/journal.pone.0076097

**Published:** 2013-10-01

**Authors:** Ryan C. Smith, Christopher Kizito, Jason L. Rasgon, Marcelo Jacobs-Lorena

**Affiliations:** 1 Department of Molecular Microbiology and Immunology, Malaria Research Institute, Johns Hopkins Bloomberg School of Public Health, Baltimore, Maryland, United States of America; 2 Department of Entomology, Center for Infectious Disease Dynamics and the Huck Institutes of the Life Sciences, Pennsylvania State University, University Park, Pennsylvania, United States of America; Centro de Pesquisas René Rachou, Brazil

## Abstract

**Background:**

Genetically modified mosquitoes have been proposed as an alternative strategy to reduce the heavy burden of malaria. In recent years, several proof-of-principle experiments have been performed that validate the idea that mosquitoes can be genetically modified to become refractory to malaria parasite development.

**Results:**

We have created two transgenic lines of *Anopheles* *stephensi* , a natural vector of *Plasmodium falciparum*, which constitutively secrete a catalytically inactive phospholipase A_2_ (mPLA_2_) into the midgut lumen to interfere with *Plasmodium* ookinete invasion. Our experiments show that both transgenic lines expressing mPLA_2_ significantly impair the development of rodent malaria parasites, but only one line impairs the development of human malaria parasites. In addition, when fed on malaria-infected blood, mosquitoes from both transgenic lines are more fecund than non-transgenic mosquitoes. Consistent with these observations, cage experiments with mixed populations of transgenic and non-transgenic mosquitoes show that the percentage of transgenic mosquitoes increases when maintained on *Plasmodium* -infected blood.

**Conclusions:**

Our results suggest that the expression of an anti-*Plasmodium* effector gene gives transgenic mosquitoes a fitness advantage when fed malaria-infected blood. These findings have important implications for future applications of transgenic mosquito technology in malaria control.

## Introduction

The ability to genetically modify mosquito vectors of malaria continues to be an attractive approach to malaria control. Synthetic peptides, effector proteins, and methods to increase the mosquito immune response have each been utilized to confer anti-*Plasmodium* resistance in proof-of-principle laboratory experiments for malaria transmission [1–4]. More recently, this approach has been further validated with the human malaria parasite, *P. falciparum*, to impair transmission using the natural host-pathogen combination [5–8].

Despite these advances, previous experiments have suggested that transgenic mosquitoes may have reduced fitness associated with the transgene [9,10]. Further evidence suggests that these reduced fitness costs may be associated with the regulatory components of the transgene or in combination with recessive mosquito genes surrounding the transgene insertion [11-13], thus highlighting the importance of identifying transgenic mosquito lines with minimal effects on mosquito fitness for any future implementation strategies that require the introgression of the transgene into a wild population. Additional evidence suggests that *Plasmodium* infection causes reduced mosquito fecundity [14–16], presumably through trade-offs between resources devoted to reproduction and the immune response to the parasite [17].

In agreement with these principles, we have previously demonstrated that transgenic mosquitoes expressing the anti-*Plasmodium* peptide SM1 display a significant fitness advantage when maintained on *P. berghei*-infected blood [18]. However, one caveat in interpreting these results is that *An. stephensi* is not a natural vector for *P. berghei*, and that this artificial combination does not accurately depict natural host-pathogen interactions. Moreover, the SM1 peptide used in these experiments does not significantly inhibit *P. falciparum* ookinete development [19]. To investigate whether a transgene that limits *P. falciparum* development could confer a selective advantage when expressed from its natural vector, we developed transgenic *An. stephensi* that express a catalytically inactive phospholipase gene (mPLA_2_) under the control of a constitutive midgut promoter. These transgenic mosquitoes effectively inhibit human and rodent malaria parasite development and display a significant fitness advantage over wild type mosquitoes in cage experiments using *P. falciparum*-infected blood.

## Results

### Generation of transgenic *An*. *stephensi*

**Figure 1:**
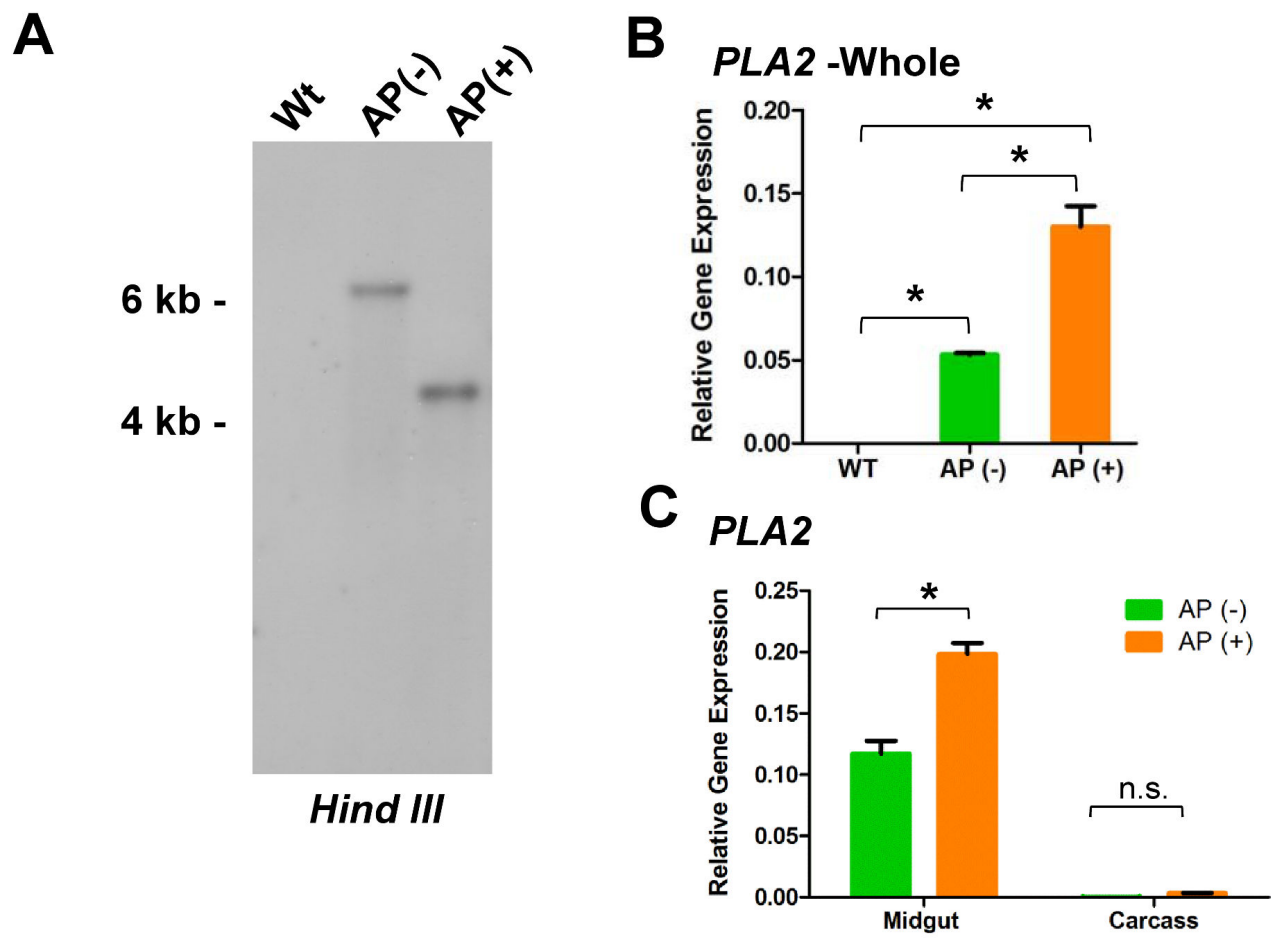
Characterization of mPLA_2_-transgenic mosquito lines. (A) Two individual *An*. *stephensi* transgenic lines were created using the previously described *piggyBac*[3 × P3-EGFP(AgPerPLA2m)] construct [22]. DNA from non-transgenic parental mosquitoes (wt) or from mosquitoes of each of the transgenic lines [AP(-) and AP(+)] was digested with HindIII, fractionated by gel electrophoresis and blotted, followed by hybridization with a *piggyBac* probe. Quantitative RT-PCR was used to measure PLA_2_ mRNA abundance relative to ribosomal protein S7 mRNA in two independent biological replicates of whole mosquitoes (B) and in specific tissues (C) obtained from colony cages of both transgenic lines. Significant differences (*P*<0.05) are denoted by an asterisk as measured using a one-way ANOVA or Student’s *t*-test. n.s.: not significant.

Previous experiments have identified that a snake venom phospholipase (PLA_2_) can limit the association of invading *Plasmodium* ookinetes with the mosquito midgut epithelium [20]. Transgenic mosquitoes expressing an active PLA_2_ enzyme significantly impair *P. berghei* development in the mosquito [2,21]. Yet, expression of the active enzyme results in significant fitness costs [11,21]. However, when rendered catalytically inactive, PLA_2_ expression in the mosquito retains its anti-*Plasmodium* effects without noticeable fitness constraints [22]. For this reason, the catalytically inactive mutant PLA_2_ (mPLA_2_) construct (*piggyBac* [3XP3-EGFP; AgPer1-mPLA_2_]) was used to generate two lines of transgenic *An. stephensi* under the control of a constitutively-expressed midgut-specific promoter (AgPer1) [21]. The two transgenic lines were initially distinguished from each other by the presence or absence of GFP fluorescence (GFP is the transformation marker) in the anal papillae [referred to as anal papillae negative, AP (-) or as anal papillae positive, AP (+)]. Southern blot analysis confirmed that the two insertions occurred at different chromosomal locations (Figure 1A).

Expression of mPLA_2_ was detected only in transgenic *An. stephensi*, with significant differences of transgene expression between the AP (-) and AP (+) transgenic mosquitoes (Figure 1B) that can likely be attributed to position effects as a result of surrounding chromatin context. Further experiments verified that mPLA_2_ expression under the constitutive *Ag*Per1 promoter is midgut-specific (Figure 1C).

### Transgenic *An*. *stephensi* inhibit *Plasmodium* development

**Figure 2:**
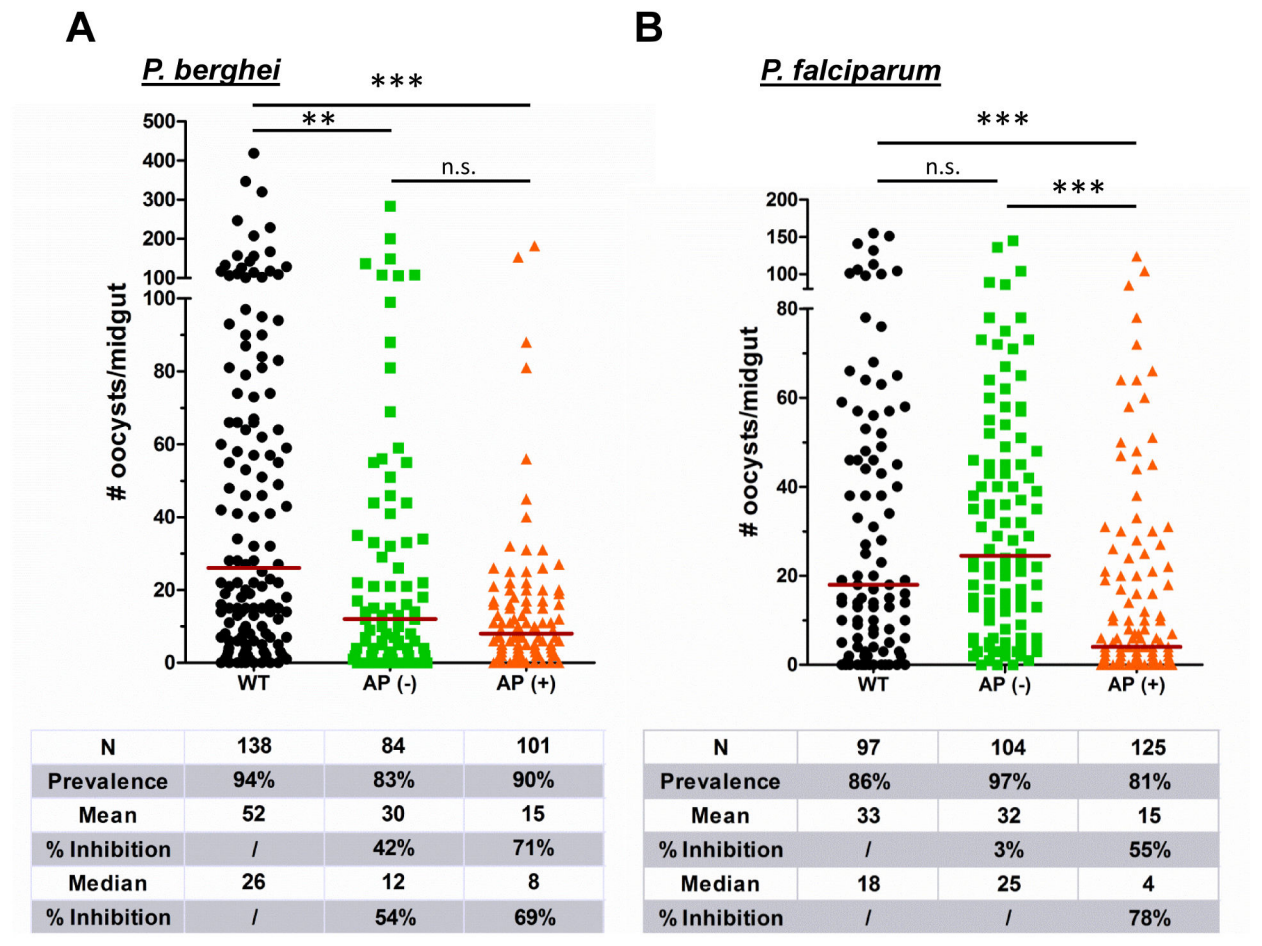
mPLA_2_-transgenic mosquitoes inhibit *Plasmodium* oocyst formation. Wild type and transgenic *An*. *stephensi* lines were fed on *P. berghei*-infected mice (A) or on *P. falciparum* gametocyte cultures (B). Data from three independent experiments were pooled in both (A) and (B). Transgenic mosquitoes obtained from colony cages (mixed homozygous and homozygous for the transgene) were used for *P. berghei* blocking experiments, while transgenic mosquitoes hemizygous for the transgene were used for *P. falciparum* experiments. The prevalence of infection (% infected/total) was lower for both transgenic lines in experiments with *P. berghei*, but only the AP (+) line with *P. falciparum*. This reduction in prevalence was analyzed by Chi-squared analysis and determined to be not significant. The horizontal red line denotes the median oocyst number for each experiment. N: number of mosquitoes analyzed. Results were analyzed using by a Kruskal-Wallis non-parametric one-way ANOVA and Dunn’s post-test to determine significance. Significant differences are denoted by asterisks: **=(*P*<0.01), ***=(*P*<0.001), n.s.: not significant.

Compared to wild type *An. stephensi*, both transgenic lines significantly impaired *P. berghei* development (Figure 2A), consistent with previously published data for the PLA_2_ transgene [2,21,22]. In contrast, when fed with human malaria parasites, only the AP (+) transgenic line significantly inhibited *P. falciparum* parasite development (Figure 2B), as the AP (-) line had no effect. We measured a small decrease in infection prevalence for both transgenic lines with *P*. *berghei* and the AP (+) line with *P*. *falciparum* (Figure 2), but the results were not significant when evaluated by Chi-squared analysis. The difference in inhibitory properties against *P*. *falciparum* may be related to the higher levels of PLA_2_ expression in the AP (+) line, but the mechanistic basis for the differences in inhibition between *Plasmodium* species is presently unclear.

### mPLA_2_-transgenic mosquitoes have a fitness advantage when maintained on *P. falciparum*-infected blood

**Figure 3:**
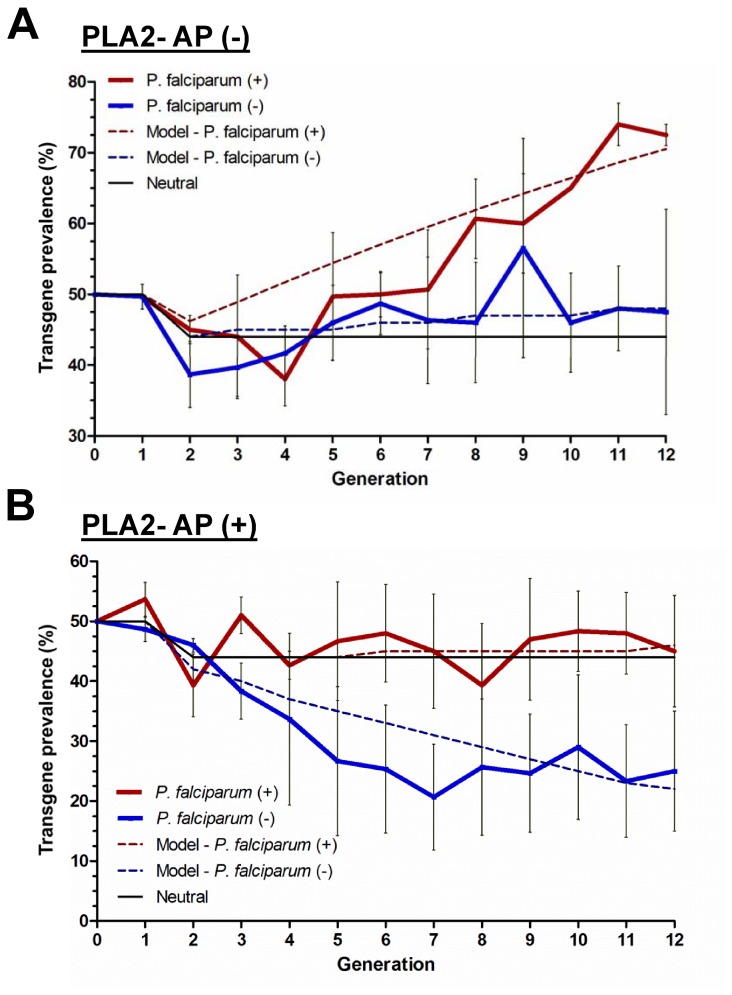
Transgenic mosquitoes have a fitness advantage when fed on *P. falciparum*-infected blood. Population cages were started with equal numbers of wild type *An*. *stephensi* and PLA_2_-transgenic mosquitoes hemizygous for the transgene and maintained either on *P. falciparum*-infected or non-infected human blood. Results reported for the AP (-) transgenic line (A) and the AP (+) transgenic line (B). Transgene prevalence was monitored in cage populations maintained on *P. falciparum*-infected (red) or non-infected (blue) blood for twelve generations. For each experimental condition (infected or non-infected blood), the average transgene prevalence of three independent experiments at each generation is denoted with error bars accounting for the standard error of the mean (SEM) between experiments. Dashed lines represent the best-fit model results. The solid black line represents the expected outcome if there is no cost or benefit to being transgenic.

Cage experiments were performed to determine whether the AP (-) and AP (+) transgenic lines have a fitness advantage over wild type mosquitoes. For these experiments, starting populations consisting of equal numbers of wild type and mPLA_2_-transgenic mosquitoes were maintained on P. falciparum-infected blood or as a control, on normal human blood (procedures outlined in Figure S1 in File S1). The transgene prevalence was monitored for both transgenic lines for 12 generations and in three independent experiments. Consistent with the results of Figure 2B, P. falciparum oocyst formation was only inhibited in cage experiments for the AP (+) transgenic line (Figure S2 in File S1). We found that both transgenic lines displayed a fitness advantage (measured by an increase in the percentage of transgenic mosquitoes) when maintained on P. falciparum-infected blood (Figure 3). Surprisingly, despite any noticeable effects on parasite development in the AP (-) line, transgene prevalence was much higher (~70%) when maintained on infected blood compared to mosquitoes maintained on non-infected blood (Figure 3A). According to best-fit model simulations, both the AP (-) and AP (+) lines exhibited a 12% fitness benefit when fed on parasite-infected b

lood (Figure 3).

In control experiments where mosquitoes were maintained on non-infected human blood, transgene prevalence in the AP (-) line remained close to Hardy-Weinberg equilibrium (Figure 3A). In contrast, the AP (+) transgenic line appears to bear a significant fitness load, as evidenced by the reduced transgene prevalence from the predicted neutral model when maintained on non-infected blood (Figure 3B). Given that transgenic mosquitoes in the AP (+) line exhibited significant fitness costs to being transgenic, while the AP (-) line had no measurable fitness effects (Figure 3), it is likely that the increased PLA_2_ expression (Figure 1B) or position effects associated with the transgene insertion contribute to the fitness costs of the AP (+) line. Previous experiments with the same mPLA_2_ construct in a heterologous mosquito did not produce measurable fitness costs [22], suggesting that fitness differences between mosquito lines may be related to the insertion of the transgene.

An additional parameter of transgene inheritance was evaluated by measuring the proportion of mosquitoes that were heterozygous or homozygous for the transgene in the last generation of a cage experiment using PCR genotyping (Figure S3 in File S1). Based upon Hardy-Weinberg analyses, the AP (-) transgenic line exhibited a classic overdominance (cost associated with being homozygous) for the transgene when maintained on non-infected blood, as shown by a significant decrease in the proportion of homozygous individuals (Figure S3 in File S1). However, these costs appear to be alleviated when AP (-) transgenic mosquitoes were fed on *P*. *falciparum* gametocytes (Figure S3 in File S1). In contrast, the AP (+) transgene showed no effect when mosquitoes were maintained on human blood, while showing a significant increase in the proportion of mosquitoes homozygous for the transgene (underdominance) when they were maintained on infectious human blood (Figure S3 in File S1). While the genotyping data were collected from only a single generation of our cage experiments, they suggest that for both transgenic lines inheritance of the mPLA_2_ transgene is favored when mosquitoes are fed infectious *P*. *falciparum* blood.

### Transgenic mosquitoes are more fecund than wild type when maintained on *P. falciparum*-infected blood

**Figure 4:**
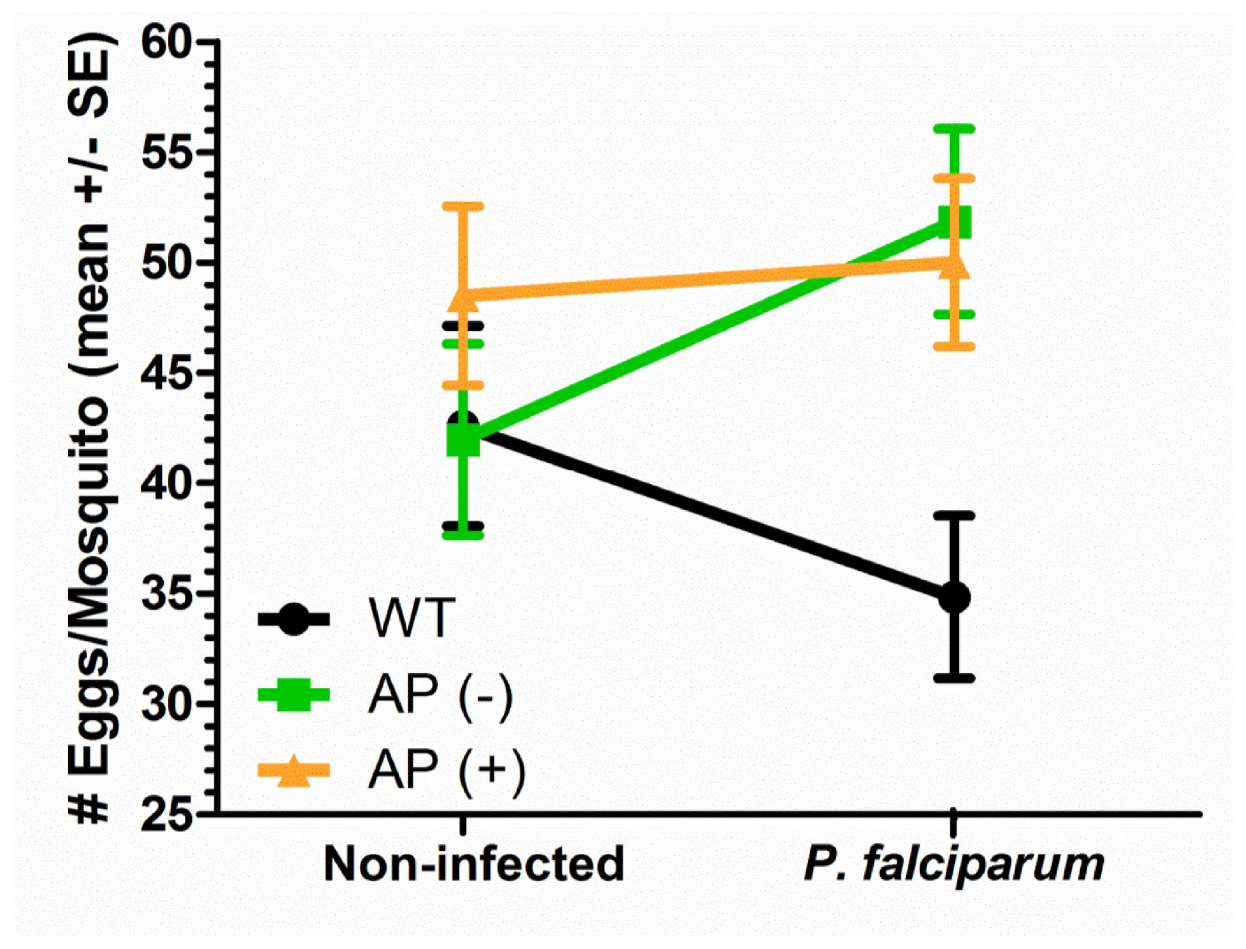
mPLA_2_-transgenic females are more fecund than their non-transgenic counterparts when fed *Plasmodium* -infected blood. Transgenic *An*. *stephensi* mPLA_2_ lines obtained from colony cages and wild type *An*. *stephensi* were fed non-infected human blood (control) or blood containing *P. falciparum* gametocytes and three days post-blood meal, the number of eggs oviposited by individual females was counted. The interaction line chart shows the mean number of eggs oviposited per female with error bars representing the SEM. Data from two independent experiments were pooled and analyzed with a two-way ANOVA and Bonferroni post-test. The total number of female mosquitoes analyzed for each experimental condition is as follows: non-infected [WT = 39, AP (-) = 39, AP (+) = 38] and *P. falciparum*-infected [WT = 48, AP (-) = 43, AP (+) = 47]. Oviposited egg numbers were not significantly different when mosquitoes were fed non-infected blood, but the AP (-) and AP (+) transgenic lines produced significantly more eggs than wild type mosquitoes when fed infectious *P. falciparum* blood (*P*<0.05 and *P*<0.01 respectively).

To identify contributing factors to the increase in transgene prevalence, the fecundity of each line was estimated by measuring the number of oviposited eggs. Both transgenic lines were significantly more fecund than wild type mosquitoes when fed on an infectious blood meal (Figure 4). For the AP (-) transgenic line, female oviposition was similar to wild type mosquitoes when maintained on non-infected blood, but significantly increased following an infectious *P*. *falciparum* blood meal. Female mosquitoes of the AP (+) line also displayed a small increase in the number of eggs produced when fed upon a parasite-containing blood meal (Figure 4). In addition, we found a small (but not significant) decrease in the numbers of oviposited eggs when wild type *An*. *stephensi* were maintained on *P*. *falciparum*-infected blood (Figure 4) in agreement with previous reports [14,18].

Together, these results suggest that the infection status of the mosquito can have large impacts on fecundity, and may explain in part the increase in transgene prevalence in the cage experiments when maintained for multiple generations on *P. falciparum*-infected blood (Figure 3). However, there is no apparent relationship between resistance to the parasite and fecundity. While both of our mPLA2 transgenic lines show a significant increase relative to wild type in the number of oviposited eggs when fed *P. falciparum*-infected blood, only the AP (+) measurably restricts *P. falciparum* development.

### Identification of transgene insertion sites

We explored the possibility that the insertion position of the *piggyBac* element into the *An. stephensi* genome might impact the fitness of our transgenic mosquito lines. To identify the genomic position of each insertion, we performed splinkerette PCR (spPCR) [23,24] on genomic DNA from both transgenic lines.

**Figure 5:**
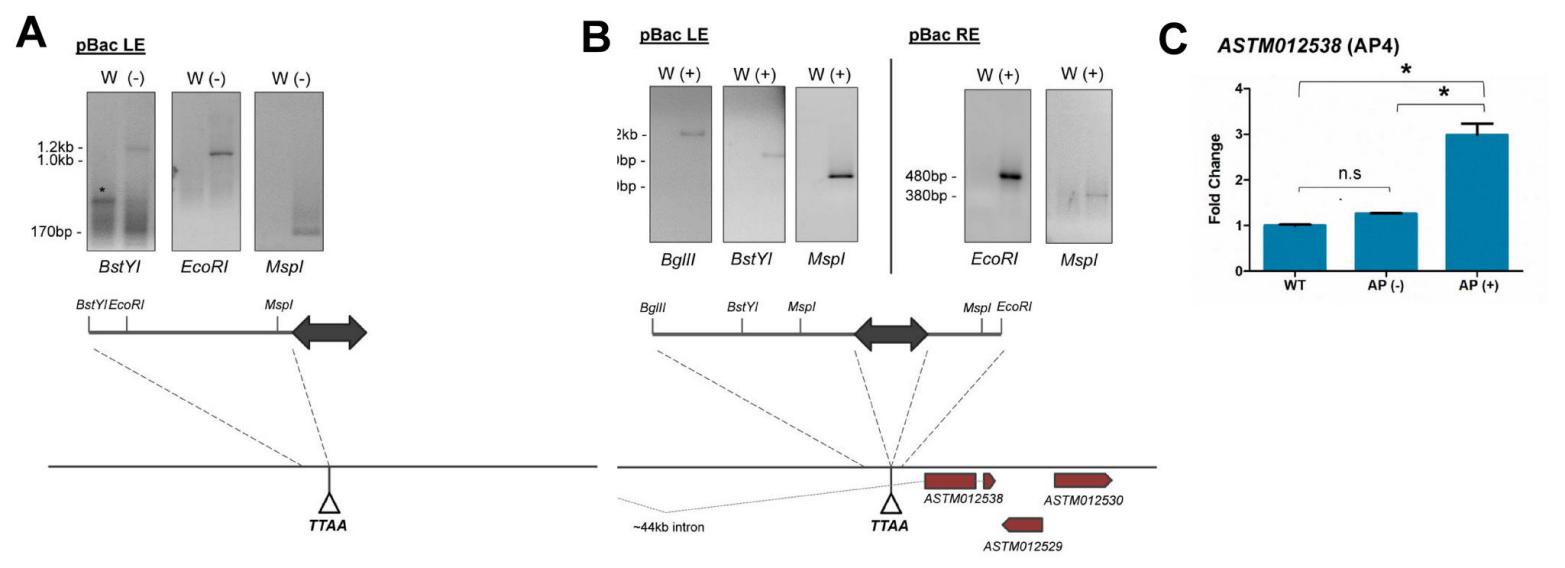
Identification of transgene integration sites. Genomic positions of transgene integration were identified for the AP (-) (A) and AP (+) (B) transgenic mosquito lines using splinkerette PCR. Identification was based on multiple PCR products for each end of the transgene, suggesting that each transgene integrated at a single, canonical *piggyBac* integration site. Using the *An*. *stephensi* genome database, 20 kb of sequence flanking each end of the insertion was identified. No annotated transcripts are present around the AP (-) integration site, while for the AP (+) line integration occurred at a highly transcribed locus (B). To determine whether the transgene influences surrounding gene function, the most proximal gene to the AP (+) integration site, a predicted AP4 transcription factor (ASTM 012538), was analyzed by qRT-PCR in (C). For each sample, the relative AP4 transcript abundance was measured in whole mosquitoes using rpS7 as an internal reference, and the abundance in wild type *An*. *stephensi* (WT) was used to calculate fold change. The abundance of the AP4 transcript was significantly higher in AP (+) transgenic mosquitoes using a one-way ANOVA with a Tukey post-test. pBac LE: left end of the *piggyBac* inverted repeat; pBac RE: right end of the *piggyBac* inverted repeat; W: DNA from wild type mosquitoes; (-): DNA from AP(-) mosquitoes; (+): DNA from AP(+) mosquitoes; TTAA: *piggyBac* canonical insertion site.

Multiple PCR fragments were recovered using different restriction digests, confirming the presence of a single *piggyBac* integration for each transgenic line and validating the Southern Blot results (Figure 1A). For the AP (-) line, PCR fragments were only obtained for the genomic region flanking the *piggyBac* left inverted terminal repeat (ITR) (Figure 5A). Although we were not able to identify sequences flanking the *piggyBac* right ITR, the canonical TTAA target site identified for the left end sequence suggests that the integration occurred through a traditional cut-and-paste mechanism. Using the left end flanking sequence, BLAST analysis revealed that this sequence corresponded to a ~40kb region (~20kb flanking each side of the TTAA integration site) within *An*. *stephensi* (Scaffold0081: 584154-625032). *In silico* analysis of the region surrounding the AP (-) insertion did not reveal any annotated coding sequences, suggesting that the *piggyBac* element inserted into a poorly transcribed and possibly heterochromatic region of the *An*. *stephensi* genome (Figure 5A). This may account for the lower PLA2 expression from the transgene in the AP (-) line (Figure 1B and 1C), and the minimal effects of the transgene on mosquito fitness (Figure 3A).

Flanking sequences were obtained for both ends of the AP (+) integration site, revealing canonical *piggyBac* TTAA target site duplications (Figure 5B). BLAST analysis mapped the AP (+) insertion to a single genomic position (Scaffold0023: 1422154-14262700). Further analysis determined that the AP (+) insertion occurred in close proximity to several annotated genes, based on preliminary analysis of *An. stephensi* EST libraries and by homology to the *An. gambiae* genome assembly (Figure 5B).

Transgene insertion in the AP (+) line causes increased expression of an AP4-like transcription factor

The *piggyBac* insertion in a gene-rich region in the AP (+) transgenic line (Figure 5B) raised the possibility that the transgene integration may have altered expression of these genes. *In silico* analysis using VectorBase [25] indicated that the *piggyBac* element inserted within a large ~44kb intron between the third and fourth exons of the annotated ASTM 012538 transcript. Homology-based BLAST analysis suggests that ASTM 012538 likely encodes an AP4-like transcription factor, referred to as *cropped* in *Drosophila* , with a yet unknown function.

To determine whether the insertion altered ASTM 012538 (or *As*AP4) transcript abundance, we performed qRT-PCR on cDNA prepared on adult female mosquitoes from wild type *An. stephensi* and from both transgenic lines (Figure 5C). Although we did not examine *As*AP4 gene expression in other life stages, *As*AP4 expression was more than three-fold higher in the AP (+) line than in wild type or AP(-) adult female mosquitoes (Figure 5C). These results suggest that the increased *As*AP4 transcript abundance may contribute to the observed fitness costs associated with the AP (+) transgenic line (Figure 3B), but validation of this hypothesis requires further experimentation.

## Discussion

The transmission of malaria is completely dependent on the successful development of the parasite in mosquitoes of the genus *Anopheles* . For several years, research has focused on interrupting the *Plasmodium* parasite life cycle within its mosquito host through a variety of approaches, including the genetic modification of mosquitoes to make them refractory to the parasite. Our data demonstrate that transgenic *An. stephensi* mosquitoes expressing a mutant PLA_2_ construct under the control of a constitutively active midgut promoter can impair both *P. berghei* and *P. falciparum* development. While previous experiments have illustrated that transgene expression of PLA_2_ can inhibit the rodent and avian malaria lifecycles in the mosquito [2,21,22], we establish for the first time that PLA_2_ transgene expression can also inhibit development of a human malaria parasite. Importantly, our cage experiments are the first to demonstrate that transgenic mosquitoes have a fitness advantage using a natural mosquito-parasite combination. However, the precise mechanism by which this occurs is unclear as both transgenic lines have an advantage when infected by *P. falciparum*, yet only the AP (+) line significantly blocks *P. falciparum* development.

Despite the reduction in oocyst numbers in our transgenic lines, we did not see a significant decrease in the infection prevalence in our experiments with *P. berghei* and *P. falciparum*. Although in contrast to previous experiments with PLA_2_ [2,20-22], a reduction in oocyst numbers without a significant impact on infection prevalence would likely have little effect on natural malaria transmission models. Therefore, it is critical to identify transgenes that reduce infection prevalence for future transgenic mosquito field applications.

Although PLA_2_ has been used extensively in transgenic mosquitoes, very little is known regarding the mechanisms of its anti-*Plasmodium* action. Experiments suggest that PLA_2_ inhibits *Plasmodium* ookinete midgut invasion [20], and that this is independent of enzymatic function [22]. In our transgenic lines PLA_2_ is constitutively expressed through the regulatory regions of the AgPer1 promoter [21]and previous experiments suggest that mPLA_2_ is secreted into the midgut lumen following blood feeding where it interferes with ookinete invasion [21,22]. We observed differences in mPLA_2_ effector gene expression between our transgenic lines even though they carry the same construct, providing evidence for position-effects similar to those previously observed in other transgenic studies [26-28]. Both transgenic lines strongly inhibit *P. berghei* development, yet only the higher-expressing line (AP+) inhibits transmission of human malaria parasites. One possible explanation is that higher levels of mPLA_2_ expression may be needed to effectively block *P. falciparum*. While these differences in inhibition between malaria parasites are not completely understood, these data are the first (to our knowledge) to report differences between transgenic lines in their ability to inhibit rodent and human malaria parasites. These differences emphasize the importance of evaluating multiple transgenic lines in future studies.

There is evidence that mosquitoes naturally resistant to the parasite have significant fitness costs, which are likely due to competition for resources devoted to the anti-parasite immune response and reproduction [17,29,30]. Consistent with these results, transgenic *An. stephensi* that over-express the transcription factor *Rel2* in response to a *Plasmodium* -infected blood meal show reduced fecundity [6]. In contrast with these results, previous work using the rodent malaria model has demonstrated that genetically engineered parasite resistance using an anti-*Plasmodium* effector gene (SM1) may allow the mosquito to devote more resources towards egg production [18]. Our data suggests that a similar mechanism exists when transgenic mosquitoes expressing an anti-*Plasmodium* effector gene (mPLA_2_) are infected with *P. falciparum*. However, since only one of our transgenic lines significantly inhibited parasite development, it is unclear what role parasite resistance may have contributed to modulating mosquito fitness in our cage experiments. mPLA_2_ expression might improve the conversion of resources from an infected blood meal into developing eggs through an unknown mechanism.

Several groups have described fitness costs associated with transgenesis [9–12], yet we provide the first evidence that these effects may be mediated by the aberrant regulation of a gene around the transposon insertion site. Conceivably, alteration of mosquito physiology and fitness could be attributed to such changes in gene expression. These considerations lend support to technologies that involve site-specific integration of transgenes [8,27]. Moreover, our results emphasize the importance of selecting a favorable integration site for docking sequences when using site-specific integration of transgenes.

In summary, our experiments demonstrate for the first time that transgenic anopheline mosquitoes exhibit a fitness advantage over non-transgenic mosquitoes in an epidemiologically relevant model using the human malaria parasite, *P. falciparum*, and its natural vector, *An. stephensi*. While these experiments were performed under ideal laboratory conditions, presumably a similar transgene with comparable fitness levels to wild type mosquitoes may promote its spread into field populations. However, one major caveat is that the frequency that a mosquito encounters an infectious blood meal is dramatically lower in the field than in our experiments, making it unlikely that the introgression of the transgene into a wild population would occur on this basis alone. Rather, introgression would have to rely on a gene drive mechanism to establish the transgene. Once established, transgenic mosquitoes that interfere with parasite development should reduce the incidence of malaria transmission and contribute to malaria eradication from the target area.

## Materials and Methods

### Ethics statement

This project was carried out in accordance with the recommendations of the Guide for the Care and Use of Laboratory Animals of the National Institutes of Health. The animal protocol was approved by the Animal Care and Use Committee of the Johns Hopkins University (protocol number M009H58). Anonymous human blood used for parasite cultures and mosquito feeding was obtained under IRB protocol NA 00019050 approved by the Johns Hopkins School of Public Health Ethics Committee. The institutional review board waived the need for written informed consent from the participants (blood donors).

### Embryo Microinjection and Mosquito Rearing

Two independent *An. stephensi* transgenic lines [AP (-) and AP (+)] were obtained from embryo microinjections with a mixture of a *piggyBac*[3 × P3-EGFP(AgPerPLA _2_m)] donor plasmid (0.5 mg/ml) and *piggyBac* helper plasmid (0.3 mg/ml) as previously described [1,20-22,31]. Surviving adults were crossed to wild type *An*. *stephensi* in small pools and transformants were selected by screening progeny for the presence of GFP. The individual lines were isolated based on their differential patterns of GFP expression in the anal papillae of transgenic larvae and maintained as a homozygous population. Mosquitoes were maintained on 10% sucrose at 27 °C and 80% relative humidity with a 14/10 h light/dark cycle as previously described [17].

### *Plasmodium berghei* Infections

For *P. berghei* infections, Swiss Webster mice were infected with *P. berghei* ANKA-GFP [32] parasites according to standard protocol [33]. The infectivity of each mouse was determined by measuring parasitemia and those mice with 1-2 exflagellations/field under a 20x objective were used for mosquito infections. Wild type *An. stephensi* or mosquitoes from each transgenic line (AP (-) or AP (+)) were fed on anaesthetized mice and blood-fed mosquitoes were maintained at 19° and 80% humidity. At 10 days post-infection, parasite infection was quantified by measuring oocyst numbers. Midgut dissections were performed in 1X PBS and stained with 0.2% mercurochrome to visualize oocyst numbers using a compound microscope.

### *Plasmodium falciparum* Infections

*P. falciparum* infections were performed by diluting mature NF54 gametocytes to 0.3% gametocytemia and mosquitoes were fed using an artificial membrane feeder. The reported inhibition experiments (Figure 2) were performed with hemizygous transgenic mosquitoes [AP (-) and AP (+)] used to establish each cage experiment and age-matched wild type *An*. *stephensi* females. Following infection, blood-fed mosquitoes were maintained at 25° and 80% humidity. Oocyst numbers were determined by dissecting midguts in 1X PBS 8 days post-infection, staining with 0.2% mercurochrome and counted with the aid of a compound microscope.

*P. falciparum* infections carried out during the course of the cage experiments were performed in similar fashion using mature NF54 gametocytes diluted to a 0.3% gametocytemia for feeding with artificial membrane feeders. Infections were monitored between 7 and 9 days post-feeding (after oviposition) by screening surviving adults for the presence of GFP fluorescence to separate transgenic and non-transgenic individuals within each experimental cage. Midgut dissections were performed in 1X PBS, stained with 0.2% mercurochrome, and the number of *P. falciparum* oocysts was determined with a compound microscope. To evaluate Plasmodium development in AP (-) and AP (+) transgenic mosquitoes, oocyst numbers were compared at each generation between transgenic and non-transgenic individuals from the same cage (summarized in Figure S2 in File S1).

### Transgenic Cage Experiments

The transgenic cage experiments were performed essentially as described by Marrelli et al. [18] with slight modifications. To initiate each cage experiment, virgin females from each transgenic line homozygous for the transgene were crossed with wild type *An. stephensi* males to produce offspring hemizygous (one copy) for the transgene. The resulting GFP^+^ progeny were used to seed four initial population cages each containing ~250 virgin wild type *An. stephensi* males from our primary mosquito colony. Two of these cages were then seeded with ~250 virgin hemizygous transgenic AP (-) females and two with ~250 virgin hemizygous transgenic AP (+) females. For each transgenic line, replicate cages were then maintained on either *P*. *falciparum* gametocyte-containing blood (experimental) or non-infected human blood (control) to obtain eggs for the next generation (outlined in Figure S1 in File S1).

Three days after blood feeding oviposition cups were placed into each cage overnight to collect eggs on a moist filter paper. The following day, oviposited eggs were transferred to plastic trays containing distilled water, a small amount of ground fish food (Tetramine), and placed into dedicated insectaries maintained at 27 °C, 80% relative humidity, and a 14/10 h light/dark cycle. Resultant larvae were maintained at low-density and percentage of transgenic mosquitoes was assessed by randomly sampling ~100 larvae from each experimental condition (experimental and control) for the presence of GFP. Adult mosquitoes were collected into cages and maintained on 10% sucrose under standard insectary conditions (as described above) until blood feeding 4-7 days post-eclosion.

In subsequent generations, mosquitoes were allowed to mate at random and cages were maintained with at least 250 female mosquitoes. Each cage experiment was carried out for a total of twelve generations and repeated in three independent biological experiments for both transgenic lines. Adult cages (experimental and control) were kept at 25°C and 80% humidity following blood-feeding to allow for the propagation of *P. falciparum* infections and to ensure that mosquitoes were reared under similar experimental conditions.

### Fecundity analysis

Age-matched adult female mosquitoes from colony cages of both transgenic lines and wild type *An. stephensi* were evaluated to identify any differences in fecundity when fed on *P. falciparum*-infected or non-infected human blood. Engorged females were separated and individually placed into sealed containers containing a moist filter paper. The number of oviposited eggs from each mosquito was determined by counting with the aid of a dissecting microscope and analyzed as previously described using a two-way ANOVA [18].

### Modeling transgene prevalence in mosquito populations

The dynamics of the prevalence of the transgene in mosquito population cages was investigated using a model previously developed [18] with minor modification. Based on Hardy-Weinberg analysis of the last generation of cage experiments, we observed both overdominant and underdominant effects depending on the transgenic line and experimental condition. Although further experiments over multiple generations are required to determine if these complex interactions are repeatable, for simplicity we excluded the effects of overdominance or underdominance in our analysis.

### Southern Blot Analysis

Five micrograms of genomic DNA was prepared from transgenic and non-transgenic *An. stephensi*, and digested with HindIII as previously described [34]. Following gel electrophoresis and transfer, the samples were hybridized with a P^32^-labeled probe complementary to the *piggyBac* left arm [21].

### Gene expression analysis (qPCR)

Tissue samples from transgenic or wild type mosquitoes were homogenized and total RNA was isolated using TRIzol (Invitrogen) according to the manufacturer’s protocol. cDNA was prepared using SuperScriptIII (Invitrogen) and gene expression was analyzed by quantitative real-time PCR using gene-specific primers as previously described [35]. qPCR results were normalized using *An. stephensi* ribosomal protein S7 as a reference and target gene expression was analyzed according to the 2^-∆∆Ct^ method [36]. Gene-specific primers sequences are as follows; *As*rpS7 (F: 5’-TGCTAACGACACGAAGACCACAAGATT -3’ and R: 5’-GGATACTTTAAACGGCTTTCTGCGTCA-3’), PLA2 (F: 5’- CAAATCAGGGATAGGATCGGGGATAAC-3’ and R: 5’-GTGACAGGATGCTCCAGCTTGTAACAC-3’), and ASTM 012538 (AP4) (F: 5’-AAGACTCGGCTACTCTCTCGCAAAACTG -3’ and R: 5’-TTCGACACTAGCTGTTGCTGTTGAGA -3’).

### Identification of transgene integration sites

To identify the *piggyBac* integrations sites for both transgenic lines, genomic DNA was isolated from fourth instar larvae and splinkerette PCR was performed as previously described [23,24] with slight modification. A list of all primer sequences used for the creation of adaptor primers and PCR amplification are summarized in Table S1 in File S1. PCR fragments were gel-purified using the Gel DNA Recovery kit (Zymo Research) and cloned into a pJet1.2 vector (Fermentas) for sequencing. For each transgenic line, recovered sequences were mapped to the *An. stephensi* genome using BLAST and analyzed for the presence of gene-coding regions using VectorBase annotations [25].

### Transgenic mosquito genotyping

To determine the genotype of transgenic mosquitoes after 12 generations of maintenance on non-infected (control) or *P. falciparum*-infected (experimental) blood, transgenic mosquitoes from each cage experiment were identified by the presence or absence of GFP fluorescence. Since GFP is a dominant marker, individual transgenic mosquitoes were analyzed by PCR to determine their genotype (ie- one or two copies of the transgene). Genomic DNA was isolated from individual GFP^+^ adult mosquitoes with a Wizard Genomic DNA Purification kit (Promega). For each transgenic line, DNA was amplified using primers that amplify across the transposon insertion site in the absence of the transgene. The presence or absence of a PCR fragment was used to distinguish between heterozygous (PCR-positive) or homozygous (PCR-negative) individuals (Figure S3 in File S1). Primer sequences used are as follows; AP (-) (F: 5’-AACTCGGCACGTTTGTTTAGATGAGG -3’ and R: 5’-CGCAGTGTTGCATTTACATTCGGTAG -3’) and AP (+) (F: 5’-GATGACAAAGGATGACCCACATCGTA -3’ and R: 5’- GTTTTTGCGACTTTATGTATCGAGGGGT-3’). To verify all individual mosquitoes genotyped as homozygous, an additional PCR reaction was prepared using the insertion-specific forward primer with a reverse primer corresponding to the *piggyBac* left end (5’-GGCGACTGAGATGTCCTAAATTGCAC -3’) to serve as an amplification control (Figure S3 in File S1). PCR reactions were performed using Phusion DNA Polymerase (NEB) with an initial denaturation of 98°C for 30 sec, followed by 40 cycles of 98°C for 10 sec, 62°C for 20 sec, and 72°C for 30 sec.

## Supporting Information

File S1 *Supporting Information.*
Figure S1, Outline of procedures used to conduct cage experiments. Figure S2, Oocyst numbers in transgenic cage experiments. Figure S3, Genotyping of PLA2 transgenic lines. Table S1, Primer sequences used for spPCR.
(PDF)
