# Supplementary material for: Transgenic Mosquitoes Expressing a Phospholipase A2 Gene Have a Fitness Advantage When Fed Plasmodium falciparum-Infected Blood
Source: PLoS One. 2013 Oct 1;8(10):e76097. doi: 10.1371/journal.pone.0076097 (PMC3788000; doi:10.1371/journal.pone.0076097)
Supplement: File S1 — Supporting Information. Figure S1, Outline of procedures used to conduct cage experiments. Figure S2, Oocyst numbers in transgenic cage experiments. Figure S3, Genotyping of PLA2 transgenic lines. Table S1, Primer sequences used for spPCR. (PDF) [file pone.0076097.s001.pdf]

Transgenic mosquitoes expressing a phospholipase A_2 gene have a fitness advantage when fed *Plasmodium falciparum*-infected blood

Ryan C. Smith[1], Christopher Kizito[1], Jason L. Rasgon[2], Marcelo Jacobs-Lorena[1]*

[1]Department of Molecular Microbiology and Immunology, Malaria Research Institute, Johns Hopkins Bloomberg School of Public Health, Baltimore, Maryland, United States of America.

[2]Department of Entomology, Center for Infectious Disease Dynamics and the Huck Institutes of the Life Sciences, Pennsylvania State University, University Park, Pennsylvania, USA.

*E-mail: mlorena@jhsph.edu

Supporting Information

Figure S1. Outline of procedures used to conduct cage experiments.

Figure S2. Oocyst numbers in transgenic cage experiments.

Figure S3. Genotyping of PLA_2 transgenic lines.

Table S1. Primer sequences used for spPCR.

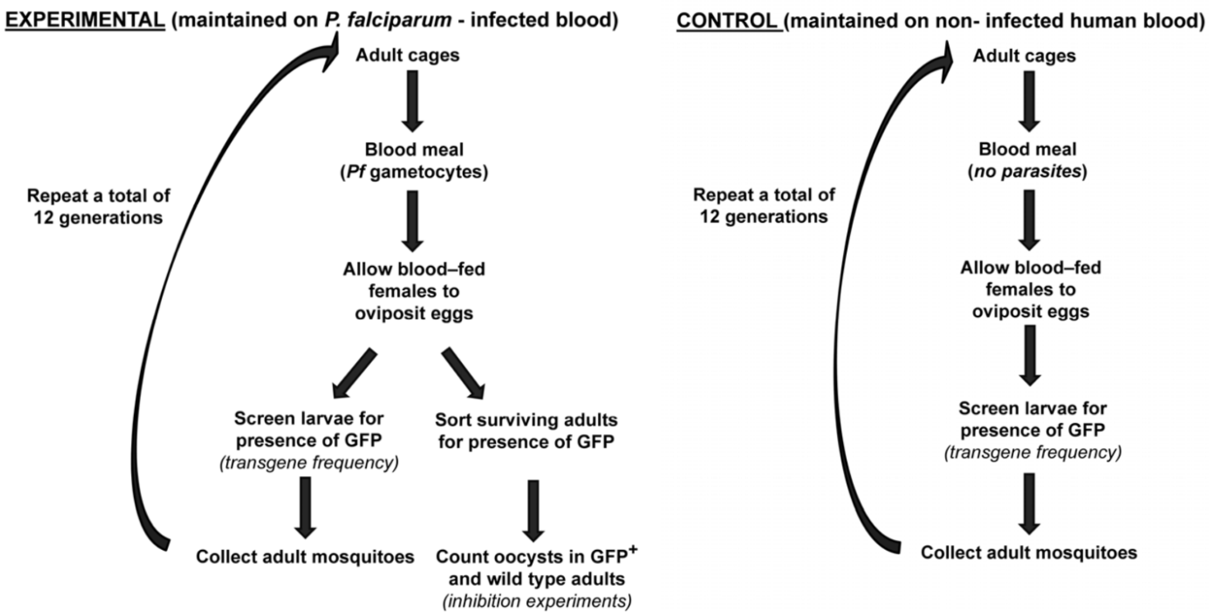

Figure S1. Outline of procedures used to conduct cage experiments.

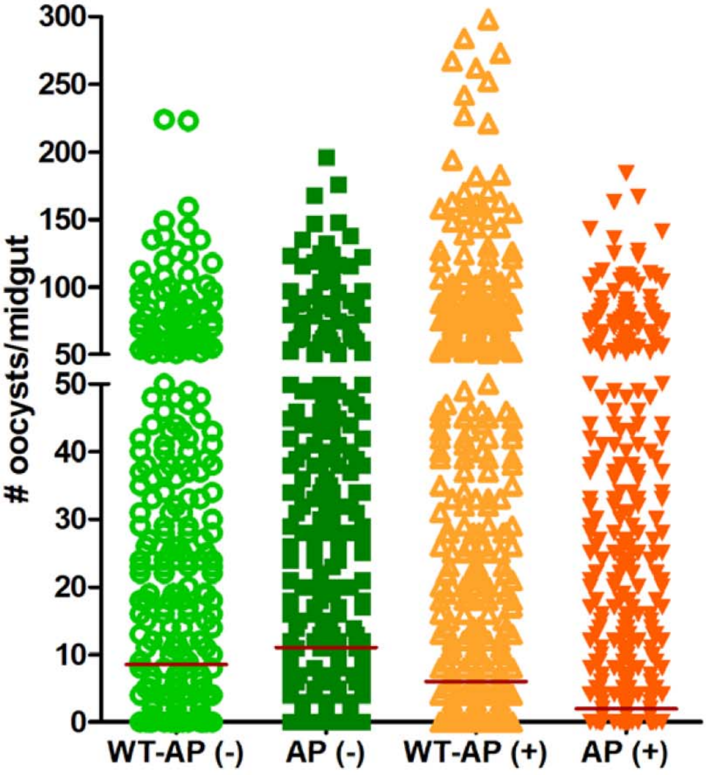

N	370	370	571	558
Prevalence	70%	71%	69%	64%
Mean	26	26	31	19
% Inhibition	/	/	/	39%
Median	8.5	11	6	2
% Inhibition	/	/	/	66%
P-value		0.9843		<.0001

Figure S2. Oocyst numbers in transgenic cage experiments. At each generation, adult female mosquitoes from each cage were individually assessed for GFP fluorescence (to distinguish transgenic from non-transgenic mosquitoes), followed by determination of *P. falciparum* oocyst numbers. Combined data for all cage experiments demonstrate that only the AP (+) transgenic line inhibited *P. falciparum* development, similar to the results in Figure 2B. The horizontal red line denotes the median value for each data set. WT-AP (-): wild type control mosquitoes for the AP (-) infections; WT-AP (+): wild type control mosquitoes for the AP (+) infections.

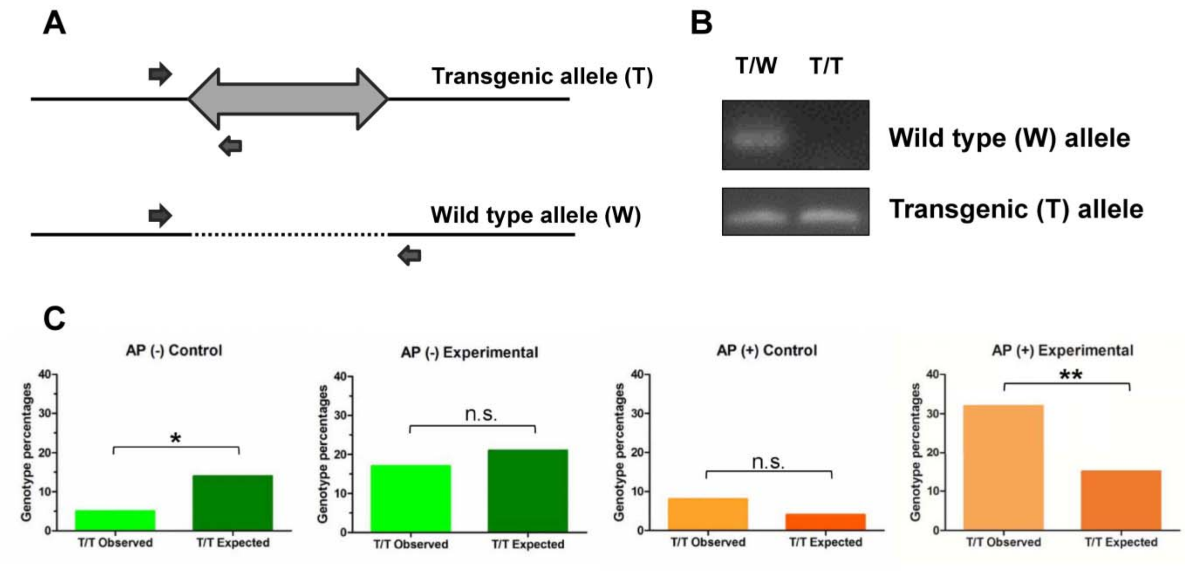

Figure S3. Genotyping of PLA_2 transgenic lines. Transgenic mosquitoes from each cage were individually genotyped by PCR to determine if the mosquito was hetero- or homozygous for the transgene. Using primers (grey arrows) that flank each insertion (A), individual GFP+ mosquitoes (containing the transgene) were genotyped by the presence/absence of the wild type allele and verified by the presence of the transgenic locus to distinguish heterozygous (T/W) or homozygous (T/T) individuals (B). Genotyping results for each transgenic cage experiment demonstrate the observed and expected percentages of mosquitoes homozygous for the transgene (C). Only the AP (+) transgenic line maintained on *P. falciparum* infected blood shows an increase in transgene prevalence as determined by Hardy-Weinberg analysis and was tested for significance by Chi-squared analysis. *= ($P<0.05$), **= ($P<0.01$), n.s.: not significant.

Table S1. Primer sequences used for spPCR.

Primer Use	Name	Sequence
**spPCR Adaptor**	Splink-Bottom (Universal)	CGAAGAGTAACCGTTGCTAGGAGAGACCGTGGCTGAATGAGACTGGTGTCGACACTAGTGG
	Splink-GATC-Top (*BstYI/BglII*)	GATCCCACTAGTGTCGACACCAGTCTCTAATTTTTTTTTTCAAAAAAA
	Splink-CGG-Top (*MspI*)	CGGCCACTAGTGTCGACACCAGTCTCTAATTTTTTTTTTCAAAAAAA
	Splink-AATT-Top (*EcoRI*)	AATTCCACTAGTGTCGACACCAGTCTCTAATTTTTTTTTTCAAAAAAA
**Adaptor primers**	Splink #1	CGAAGAGTAACCGTTGCTAGGAGAGACG
	Splink #2	GTGGCTGAATGAGACTGGTGTCGAC
***piggyBac* primers**	pBac LE #1	CAGTGACACTTACCGCATTGACAAGC
	pBac LE #2	GCGACTGAGATGTCCTAAATGCAC
	pBac RE #1	CGATATACAGACCGATAAAACACATGCGTC
	pBac RE #2	ACGCATGATTATCTTTAACGTACGTCAC
